# Apps to promote physical activity among adults: a review and content analysis

**DOI:** 10.1186/s12966-014-0097-9

**Published:** 2014-07-25

**Authors:** Anouk Middelweerd, Julia S Mollee, C Natalie van der Wal, Johannes Brug, Saskia J te Velde

**Affiliations:** 1 Department of Epidemiology & Biostatistics and the EMGO Institute for Health and Care Research, VU University Medical Center, Van der Boechorststraat 7, Amsterdam, 1081 BT, The Netherlands; 2 Department of Computer Science, VU University Amsterdam, De Boelelaan 1081, Amsterdam, 1081HV, The Netherlands; 3 Department of Psychology, VU University Amsterdam, De Boelelaan 1081, Amsterdam, 1081HV, The Netherlands

**Keywords:** Mobile phone application, Behavior change technique, Physical activity, Smartphone

## Abstract

**Background:**

In May 2013, the iTunes and Google Play stores contained 23,490 and 17,756 smartphone applications (apps) categorized as Health and Fitness, respectively. The quality of these apps, in terms of applying established health behavior change techniques, remains unclear.

**Methods:**

The study sample was identified through systematic searches in iTunes and Google Play. Search terms were based on Boolean logic and included AND combinations for physical activity, healthy lifestyle, exercise, fitness, coach, assistant, motivation, and support. Sixty-four apps were downloaded, reviewed, and rated based on the taxonomy of behavior change techniques used in the interventions. Mean and ranges were calculated for the number of observed behavior change techniques. Using nonparametric tests, we compared the number of techniques observed in free and paid apps and in iTunes and Google Play.

**Results:**

On average, the reviewed apps included 5 behavior change techniques (range 2–8). Techniques such as self-monitoring, providing feedback on performance, and goal-setting were used most frequently, whereas some techniques such as motivational interviewing, stress management, relapse prevention, self-talk, role models, and prompted barrier identification were not. No differences in the number of behavior change techniques between free and paid apps, or between the app stores were found.

**Conclusions:**

The present study demonstrated that apps promoting physical activity applied an average of 5 out of 23 possible behavior change techniques. This number was not different for paid and free apps or between app stores. The most frequently used behavior change techniques in apps were similar to those most frequently used in other types of physical activity promotion interventions.

## Background

Physical inactivity contributes to approximately 3.2 million deaths annually and is the fourth leading risk factor for premature death [1],[2]. Despite the fact that many people do not comply with physical activity recommendations [1],[3], smartphone applications (apps) that promote physical activity are popular: of the 875,683 active apps available in iTunes and the 696,527 active apps in Google Play, 23,490 and 17,756 were categorized as Health and Fitness [4],[5]. Therefore, it is worthwhile to study the potential of apps that aim to promote physical activity, especially because 56% of the US adults owns a smartphone [6].

Health behavior change interventions are more likely to be effective if they are firmly rooted in health behavior change theory [7]-[9]. Webb et al. [7] have noted the importance of behavior change theories in Internet-based interventions. Additionally, earlier studies have suggested that individually tailored feedback (i.e., feedback based on the user’s own characteristics [10]) and advice is more likely to be effective than generic information about physical activity [9],[11],[12].

Many advantages of using the Internet as a delivery mode apply to smartphone apps too: constantly accessible, adjustable to the needs of the user [13], able to provide (computer-) tailored feedback, large reach and interactive features. Because people carry smartphones and can access data anywhere and anytime, physical activity behavior change promotion apps offer the opportunity to provide tailored feedback and advice at the appropriate time and place. Therefore, apps offer new opportunities to deliver individually tailored interventions, including real-time assessment and feedback that are more likely to be effective.

Apps are relatively new tools in physical activity interventions and only very little research has been published to date on the content and the effectiveness of physical activity apps. It remains unclear to what extent apps differ in their relevant content and if these differences mediate effectiveness. Previous research suggests that the use of behavior change techniques to address behavioral determinants conceptualized in behavior change theory, is linked to effectiveness [14]. Therefore, it can be proposed that the presence of behavior change techniques in general and some specific behavior change techniques in particular is an indicator of potential effectiveness. Abraham and Michie [14] developed a taxonomy to identify behavior change techniques in a range of health promotion interventions. The taxonomy can be used to identify techniques or combinations of techniques that enhance effectiveness. The most frequently applied behavior change techniques in traditional interventions are goal-setting [14], prompt intention formation [14], providing feedback on performance [14], self-monitoring [14] and reviewing behavioral goals [15],[16]. A large body of work has been published using the taxonomy in health promotion interventions [7],[15]-[17], but so far, no study has been conducted with the aim to review application of behavior change techniques in apps.

Therefore, the present study aims to review apps developed for iOS and Android that promote physical activity among adults through individually tailored feedback and advice. Recent reviews have concluded that health promoting apps lack the use of behavior change theories in promoting behavior changes such as smoking cessation, weight-loss, and increased physical activity [18]-[21]. Only one earlier study focused on the use of behavior change theories in apps that target physical activity [18]. However, the authors limited their search to iTunes and excluded apps that targeted other health behaviors in addition to physical activity (e.g. apps that combined physical activity and diet information). Another limitation of their review was that it included apps that only provided information or solely used GPS-tracking to promote physical activity. In addition, the authors used a first generation iPad to download and review the apps and consequently had to exclude apps that were not compatible with this tablet. To improve upon the existing body of research on this topic, the current study reviews the use of behavior change techniques in physical activity apps available in both app stores (i.e., iTunes and Google Play) restricted to apps that utilize tailored feedback. Because previous studies reported a significant association between price and the inclusion of behavior change theories [18],[19], free and paid apps will be compared. Since we derived apps from two different online sources that differ in their acceptation policy, we additionally assessed whether the number of behavior change techniques differed between apps available in iTunes and Google Play.

## Methods

### Inclusion criteria

This review included apps that were available through iTunes and Google Play. Apps were included if they (i) were in English, (ii) promoted physical activity, (iii) followed the official recommendation of 150 minutes of moderate to vigorous physical activity per week [3], (iv) were primarily aimed at healthy adults, and (v) provided individually tailored feedback. Thus, apps that specifically focused on children, adolescents, older adults, pregnant women, unhealthy adults or individuals with disabilities were excluded because of the differences in physical activity guidelines for these groups [3]. Apps that provided feedback by showing logged statistics without feedback or without information about progress toward a personal user-set goal were also excluded.

### Search strategy

The study sample was identified through systematic searches in iTunes and Google Play. Apps from iTunes were identified between August and September 2012, and apps in Google Play were identified between November 2012 and January 2013. Because the two reviewers (AM and JM) screened the apps on different days, there was a slight variation in the number of apps offered in the app stores. During the search and screening period, iTunes updated its search strategies (on August 24, 2012), which reduced the number of apps retrieved with a specific search term. In case one of the reviewers retrieved fewer apps than the other due to this update, the results from the earlier search were included.

Search terms were based on Boolean logic and included AND combinations for physical activity, healthy lifestyle, exercise, fitness, coach, assistant, motivation, and support.

### Screening procedure

Because the screening procedure for iTunes differed to some extent from Google Play, the screening procedures are reported separately. If an app had a free version and a paid version, the free version was downloaded first. If the paid version had relevant extra features (tailored feedback or additional features not available for the free version), it was also downloaded and evaluated. This method was applied for both screening procedures. If the same version of an app was available in iTunes and in Google Play, the iTunes version was downloaded and assessed for eligibility. For both iTunes and Google Play, the identification and eligibility phases of screening were performed by two researchers (AM and JM, or AM and StV), and differences between the two reviewers were resolved by discussion and/or involving the third reviewer.

**Figure 1:**
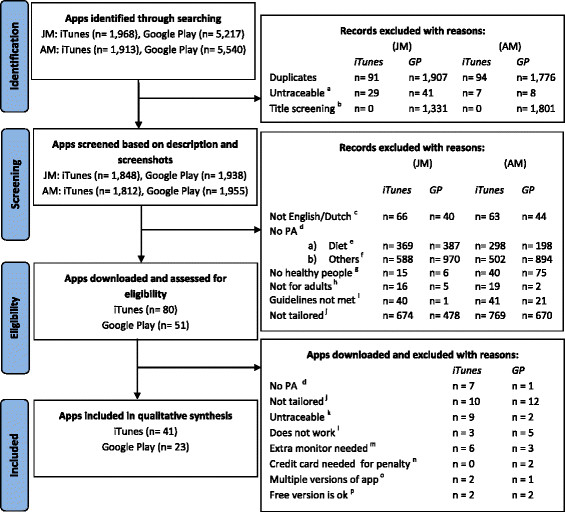
**Flow chart: schematic overview of the selection process for apps eligible for full review.** This flow chart provides a schematic overview of the selection process of eligible apps available in iTunes and Google Play (GP). The initials of the main reviewers are reported as JM and AM. ^a^Apps on the list of one researcher were untraceable for the other researcher. ^b^Apps to which the adjusted screening method had been applied and only the titles were screened. ^c^Apps that were not available in English or Dutch. ^d^The main focus of the apps was not physical activity (PA) promotion. ^e^Apps that focused on diet and weight loss. ^f^The main focus of the apps was not physical activity (PA) promotion or weight loss. ^g^Apps that targeted people with injuries or disabilities. ^h^Apps that targeted children or older adults. ^i^Apps did not follow the guidelines for physical activity. ^j^Apps that did not provide tailored feedback. ^k^Apps that were detected in the first screening step and were not available for download. ^l^After downloading the app, it did not work. ^m^An extra monitor or device was needed to receive tailored feedback. ^n^Before using the app, a credit card was needed to deduct money as a penalty if the user did not achieve self-defined goals. ^o^The same app was available under a different name but with the same features. ^p^The app had a free and a paid version, but the paid version did not have additional features.

First, the screening procedure was conducted for apps available in iTunes. Figure 1 provides a schematic overview of the decision sequence.

In the identification phase, search terms were entered in iTunes. In the screening phase, the app description and screenshots were reviewed based on the inclusion criteria. If the app appeared to be eligible, it was downloaded to an iPhone 4s smartphone and assessed for eligibility. In the eligibility phase, a reviewer explored each app by using all of its available functions.

Google—including Google Play—has a somewhat different search algorithm than iTunes. For example, it extends the search by recognizing synonyms and personal preferences, resulting in twice as many hits compared to iTunes. Therefore, the review steps were adapted for Google Play. Google Play’s search algorithms also prioritize search results, meaning that the first results listed are the most relevant and the closest to the search terms. Therefore, the adjusted screening method specified that for search terms revealing over 1,000 apps, the title, description, and screenshots of the first 100 apps were first screened carefully. If at least five out of the first 100 apps met the inclusion criteria, the next 100 apps were also screened. If one app was selected in the second group of 100 apps, the screening procedure was continued with the next 100 apps, and so on, until no apps were selected in a group of 100 screened apps. All remaining apps (AM = 1,801, JM = 1,331) were additionally screened for possible eligibility based on their title. If the title indicated possible eligibility, the app was screened for inclusion. This screening procedure was applied for eight search terms that revealed over 1,000 apps: “physical activity”, “healthy lifestyle AND fitness”, “fitness AND exercise”, “fitness AND coach”, “fitness AND motivation”, “fitness AND support”, “exercise AND support”, and “physical activity AND support”.

Figure 1 provides a schematic overview of the decision sequence for the decision sequence for Google Play apps as well. In the identification phase, search terms were entered in Google Play. In the screening phase, the app description and screenshots were reviewed based on the inclusion criteria. Apps that appeared to be eligible were downloaded to an HTC Rhyme smartphone and were fully explored by using all functions available in the app.

Apps commercially available do not provide detailed (intervention) descriptions or published study protocols, therefore an alternative approach was chosen to detect behavior change techniques in apps. Firstly, all available functions (e.g. information, chat, monitoring options, reminders and graphs) were explored by using the app for about half an hour. Secondly, the apps were running in the background for a couple days so the authors were able to read the reminders and push-up messages.

### The taxonomy

The apps were rated based on the taxonomy of behavior change techniques used in interventions [14]. This taxonomy was developed to identify potentially effective behavior change techniques used in interventions [14] and was previously used to identify behavior change techniques in interventions that aimed to increase physical activity [7],[14],[15],[22]. The taxonomy distinguished 26 behavior change techniques. Three of these techniques had low inter-rater reliability and were thus not included in the present review [14], resulting in an adapted version of the taxonomy with 23 items.

### Scoring

Each app was scored by two reviewers (AM, JM) on all 23 items of the adapted taxonomy. Each app received a score of 0–23 representing the number of behavior change techniques identified. The results were entered into an electronic database (Microsoft Access 2003). In preparation for scoring each app, the reviewers studied a coding manual and discussed each item of the taxonomy carefully. For example, self-monitoring was defined as all features helping in keeping record of the behavior (e.g. GPS-tracking, diary, accelerometer). Specific goal setting was defined if a features helps with detailed planning, the goal had to be clearly defined. Plan social support was seen as all features offering social support (e.g. possibility to link with social networking sites, chat possibilities).

The apps were scored independently, and a percentage of agreement was calculated to assess inter-rater reliability between reviewers. The percentage of exact agreement was 44%, and 91% of the scores were within a difference of 1 point. Nine percent of the apps had a disagreement of > 1 point (but with a maximum of 3 points). Subsequently, differences in interpretation were resolved by discussion.

### Extracted data

The name of the app, the name of the app producer, the date it was downloaded, the name of the app store, and the price were collected for each app in addition to the app’s score based on the number of behavior change techniques it used.

### Analyses

Means and ranges were calculated for the sum behavior change technique scores and the price of apps. Significant differences in the use of behavior change techniques (between iTunes and Google Play and between free and paid apps) and in price (between iTunes and Google Play) were assessed with Mann Whitney U tests (significance level of p < .05). To compare iTunes and Google Play, apps available in both stores were excluded, otherwise the same app would be included twice in the same analyses (once in the iTunes group, once in the Google Play group).

## Results

Due to the time differences mentioned earlier, reviewer AM detected 1,913 apps in iTunes and 5,540 apps in Google Play and reviewer JM detected 1,968 apps in iTunes and 5,217 apps in Google Play. The current review included 41 apps available in iTunes and 23 apps available in Google Play, of which 30 and 21, respectively, were free. The mean price of the paid apps was €2.06 (range €0.79-8.99) for iTunes and 1.88€ (range €0.76-2.99) for Google Play. Seven apps were available in both iTunes and Google Play for free.

The average number of behavior change techniques included in the eligible apps was 5 (range 2–8). Table 1 shows the sum score for behavior change techniques for each app. One app had a score of 8 out of 23.

**Table 1:** The number of applied behavior change techniques (BCTs) in apps

**App**	**App Store**	**Price [Euros]**	**Score BCT**
RunKeeper - GPS Track Run Walk*	Google Play	0	8
Big Welsh Walking Challenge	iTunes	0	7
GymPush	iTunes	0	7
Hubbub Health	iTunes	0	7
My Pocket Coach (a life, wellness & success coach)	iTunes	0	7
Sixpack - Personal Trainer	iTunes	0	7
Teemo: the fitness adventure game!	iTunes	0	7
fitChallenge	iTunes	0.89	6
FitCoach - powered by Lucozade Sport	iTunes	0	6
Fitness War	iTunes	0	6
Running Club	iTunes	0	6
Sworkit Pro	Google Play	0.76	6
Take a Walk Lite	iTunes	0	6
Track & Field REALTIMERUN (GPS)	iTunes	0.89	6
Withings- Lose Weight, Exercise, Sleep Better, Monitor Your Heart	iTunes/Google Play	0	6
1UpFit	iTunes	0	5
All-in Fitness: 1000 Exercises, Workouts & Calorie Counter	iTunes	8.99	5
Be Fit, Stay Fit Challenge	Google Play	0	5
Endomondo Sports Tracker	Google Play	0	5
Everywhere Run! - GPS Run Walk	Google Play	0	5
Fit Friendzy	iTunes	0	5
FitCommit - Fitness Tracker and Timer	iTunes	1.59	5
Fitocracy- Fitness Social Network, Turn Working Out	iTunes/Google Play	0	5
Healthy Heroes	iTunes	0	5
Improver	iTunes	0.79	5
Macaw	iTunes/Google Play	0	5
Make your move	iTunes	0	5
Nexercise = fun weight loss	iTunes/Google Play	0	5
Nike + Running	Google Play	0	5
Noom CardioTrainer	Google Play	0	5
ShelbyFit	iTunes	0	5
SoFit	Google Play	0	5
Strava Cycling	Google Play	0	5
Tribesports	Google Play	0	5
Walk ’n Play	iTunes	0	5
20/20 LifeStyles Online	iTunes	0	4
Croi HeartWise	iTunes	0	4
Exercise Reminder HD Lite	iTunes	0	4
Faster	iTunes	1.59	4
Fitbit Activity Tracker	iTunes/Google Play	0	4
FitRabbit	iTunes	0	4
Get Active!	iTunes	0.79	4
Get In Gear	iTunes/Google Play	0	4
Go-go	iTunes	0	4
IDoMove Work out and Win	iTunes/Google Play	0	4
Poworkout Trim & Tone	Google Play	2.99	4
SmartExercise	Google Play	0	4
CrossFitr	Google Play	0	3
FitTrack	Google Play	0	3
Forty	iTunes	0.89	3
HIIT Interval Training TimerAD	Google Play	0	3
Hiking Log- (Walking, Camping, Fitness, Workout, Hike, Pedometer Tool)	iTunes	1.79	3
Mobile Adventure Walks	iTunes	0	3
Run Tracker Pro - TrainingPeaks	iTunes	2.69	3
Running Log! PRO	iTunes	1.79	3
Softrace	Google Play	0	3
Activious	iTunes	0	2
Mean		0.46	5
Standard Deviation		1.34	1

*All apps (n = 57) are ranked by the number of applied BCTs and listed alphabetically.

**Figure 2:**
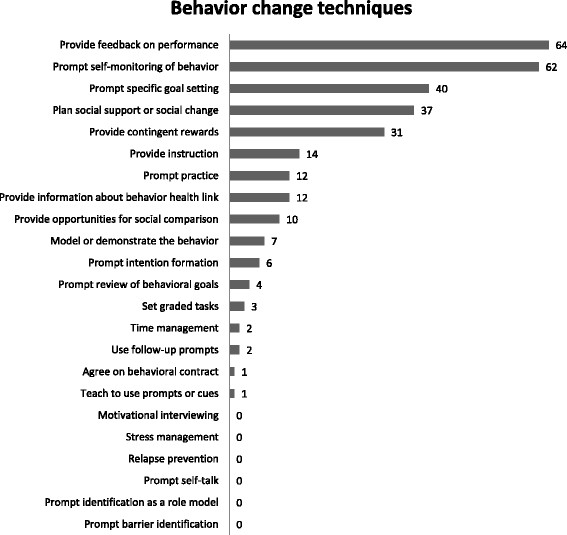
**Frequencies of the 23 behavior change techniques used in apps.** Behavior change techniques are scored using the taxonomy created by Abraham and Michie [14], ranked by the most frequently applied techniques.

Providing feedback (n = 64), self-monitoring (n = 62), and goal-setting (n = 40) were used most frequently, whereas motivational interviewing, stress management, relapse prevention, self-talk, role modeling, and prompted barrier identification were not used in any of the screened apps (Figure 2).

Free and paid apps did not differ with respect to the use of behavior change techniques (p = .18).

No differences in price were found between apps available in iTunes and Google Play (p = .14). Similarly, apps available in iTunes and Google play did not differ with respect to the number of behavior change techniques used (p = .39).

## Discussion

The current review aimed to evaluate the use of behavior change techniques in apps available through iTunes and Google Play that target physical activity and use tailored feedback, based on an established taxonomy of such techniques [14],[23]. The 64 apps included in the review used on average 5 different behavior change techniques, and none of the apps used more than 8 or less than 2. Providing feedback and self-monitoring were the most frequently used technique. At least two behavior change techniques were identified in each of the apps included in the review, which suggests that app developers attempt to use behavior change theory to some extent. However, the results also indicate that the inclusion of established behavior change techniques is far from optimal in most apps.

Studies in which behavior change theories in apps were operationalized have concluded that apps generally lack the use of theoretical constructs [18],[19],[21]. For example, West et al. [19] concluded that 1.86% of the apps in Health & Fitness included all of the factors of the Precede Proceed Model. Similarly, Cowan et al. [18] found that key constructs of behavior change theories were seldom used in apps that target physical activity. Lastly, Breton et al. [21] found a lack of adherence to evidence-based practice in apps targeting weight loss (average 3 practices, range 0–12). The findings of the present review are somewhat more favorable than earlier findings from the reviews described above. The more frequent use of behavior change techniques in the apps reviewed in the current study may be a consequence of the inclusion criteria. We only included apps that provided individually tailored feedback and excluded generic information apps, which may have resulted in the exclusion of apps that were not based on theoretical constructs. In addition, technological development in recent years may have resulted in the ability to develop more advanced app features, including the use of a wider range of behavior change techniques. Another finding that deviates from previous studies is that free and paid apps did not differ in the number of behavior change techniques used, whereas previous reviews found that price was positively associated with use of theoretical constructs [18],[19]. The differences in findings may be explained by the number of paid apps included, which was much higher in our review compared to previous reviews [18],[19].

Previous reviews that applied Abraham and Michie’s taxonomy [14] to assess the number of behavior change techniques used in non-app interventions identified on average 6–8 behavior change techniques [14],[15],[22]. Frequently used behavior change techniques are: self-monitoring, feedback on performance and goal setting [7],[15],[22]. Interventions including self-monitoring in combination with providing feedback, specific goal setting, prompt intention formation or prompt review behavioral goals showed larger effect sizes [15],[16]. Furthermore, studies reported inconclusive conclusions regarding the number of behavior change techniques that are associated with larger effects: a systematic review on web-based interventions reported that interventions that included larger numbers of behavior change techniques are more likely to be effective [7], whereas another meta-analysis suggests that the number of included behavior change techniques is not associated with a larger effect [15].

Although we found that the average number of behavior change techniques used in apps was lower than previously reported for other types of physical activity promotion, the most frequently used types of behavior change techniques used were similar [7],[15],[22]. It remains unclear if lack of theory-driven behavior change techniques in apps is due to technical difficulties or due to other factors. However, the findings of the current review, combined with our knowledge about what specific behavior change techniques have been effective in other types of behavior change interventions, suggest that apps may be an effective way to promote physical activity.

Unfortunately, little is currently known about the effect of apps on physical activity. The current review provides information about the content of apps, but future research should study how behavior change techniques can be translated into apps. Additionally, future research should examine the effectiveness of apps and which behavior change techniques or combinations of techniques are more effective.

This review indicates that apps have the potential to provide tailored feedback and to integrate behavior change techniques. Smartphones with Internet access and apps turn a cell phone into a portable personal computer. This technology offers the opportunity for ecological momentary assessment (EMA) and makes it feasible to provide timely messages based on the user’s location [23],[24]. The application of smartphones and apps in health behavior interventions are growing rapidly, however little has been published about the interventions using the new technology to provide real-time feedback [25].

A collaboration between app developers, health professionals, and behavior change experts could increase the use of behavior change techniques in apps and may open a new scale of possibilities in health promotion.

### Strengths and limitations

Scoring the content of apps is susceptible to rater bias. The level of inter-rater reliability in this review was lower than that of previous content analysis of apps [18],[19]. This study’s relatively low inter-rater reliability may be because Abraham and Michie’s taxonomy [14] was originally designed to score other behavior change interventions than smartphone app-based interventions. Applying the taxonomy to apps forced the researchers to translate the strategies into app functionalities. Following this logic, the researchers had to score each app based on what they observed. Although the researchers reviewed the apps carefully, behavior change strategies in apps may have been overlooked or interpreted differently, and some behavior change techniques may be more obvious than others. Thus, some of the behavior change techniques may be hidden in the app features and may therefore not been detected, especially follow-up prompts.

This study evaluated the use of behavior change techniques in apps that target physical activity but provides no information about the effectiveness of these apps. Further research is needed to evaluate the effectiveness of apps that promote physical activity.

The strengths of the present review include the extensive search strategy, the inclusion of both iTunes and Google Play, and the independent rating of the apps by two reviewers. Moreover, rating of the apps was not limited to apps that were free but also included retail apps. Finally, rating was done after downloading and using all of the app’s functions rather than solely using screen shots.

## Conclusions

The present study demonstrates that apps promoting physical activity applied an average of 5 behavior change techniques. There was no difference in the number of identified behavior change techniques between free and paid apps. The most frequently used behavior change techniques in apps were goal setting, self-monitoring and feedback on performance, which was similar to the ones most frequently used in other types of physical activity promotion interventions. The findings of the present study showed that apps can substantially be improved regarding the number of applied techniques.

## Abbreviations

BCT: Behavior change technique

GP: Google Play

PA: Physical activity

## Competing interests

The authors declared that they have no competing interests.

## Authors’ contributions

AM: Conducted the review, performed the analyses, drafted the manuscript and incorporated all feedback. JM: Conducted the review, provided intellectual input to the review and manuscript and approved the final version. NvdW: Provided intellectual input, provided feedback on the manuscript and approved the final version of the manuscript. JB: Provided intellectual input to the design and execution of the review and to the manuscript, provided feedback and approved the final version of the manuscript. StV: Designed the review and proved intellectual input the execution of the review, screened part of the applications, provided feedback and approved the final version of the manuscript. All authors read and approved the final manuscript.
